# Association of *HLA-B* and *HLA-DR* gene polymorphisms with rheumatoid arthritis: A cross-sectional study in Yunnan Chinese Han population

**DOI:** 10.1515/rir-2025-0022

**Published:** 2025-10-04

**Authors:** Jing Dong, Yifan Yang, Minghuang Mo, Shuang Liu, Ru Bai, Shu Li, Ruotong Zhao, Xinyu Xu, Yuqi Cheng, Jian Xu

**Affiliations:** Department of Rheumatology and Immunology, First Affiliated Hospital of Kunming Medical University, Kunming, Yunnan Province, China; Department of Rheumatology and Immunology, Qujing Second People’s Hospital, Qujing, Yunnan Province, China; Affiliated Mental Health Center & Hangzhou Seventh People’s Hospital, Zhejiang University School of Medicine, Hangzhou, Zhejiang Province, China

**Keywords:** gene polymorphism, rheumatoid arthritis, Yunnan Han nationality

## Abstract

**Objectives:**

To investigate the association between *HLA-B* and *HLA-DR* gene polymorphisms and rheumatoid arthritis (RA) in Yunnan Han population, China.

**Methods:**

A total of 246 RA patients and 259 healthy controls (HCs) were enrolled. *HLA-B/DR* genotyping was performed using high-resolution reverse polymerase chain reaction sequence specific oligonucleotide probe (PCR-SSOP). and serum anti cyclic citrullinated peptide (anti-CCP) antibody and serological indexes were detected.

**Results:**

In RA patients, the allele frequencies of HLA-DRB1*04:05:01, *04:10:01, *10:01:02 and HLA-B*15:01:01G, *40:01:01G, *40:02:01G, and *54:01:01G were significantly higher than those in the HCs (OR > 1, all *P* < 0.05). Conversely, the frequencies of HLA-DRB1*07:01:01, *14:01:01, *15:01:01:01 and HLA-B*15:01:01, *15:02:01, and *38:02:01 alleles were decreased in RA group (OR < 1, all *P* < 0.05). Haplotype analysis showed that the combination of HLA-B*15:01/DRB1*09:01, HLA-B*38:02/DRB1*08:03 and HLA-B*54:01/DRB1*04:05 was decreased in RA patients (OR < 1, all *P* < 0.05).

**Conclusions:**

The susceptibility of RA in Yunnan Han population is closely related to HLA-B/DR specific alleles and haplotypes.

## Introduction

Rheumatoid arthritis (RA) is an autoimmune disease characterized by symmetrical polyarthritis and systemic complications involving the lungs, vasculature, and other organs mediated by cytokines, immune complexes, and autoantibodies.^[1,2]^ It affects 0.5% to 1% of the global adult population, with an incidence ranging from 0.18% to 1.07% across different demographics and a female-to-male ratio of approximately 3: 1.^[3,4]^ The etiology of RA is unclear, but its pathogenesis is likely driven by a complex interplay of genetic, immunological, and environmental factors, leading to significant clinical and prognostic heterogeneity.^[5,6]^ Studies show that HLA-DRB1*04 and *10 are more frequent in RA patients than controls, while HLA-DRB1*14 is less frequent in RA patients.^[7]^

HLA-DRB1*04:05:01, DRB1*10:01:01, DQB1*04:01:01, and DPB1*02:01:02 alleles have been identified as RA risk alleles in Chinese Han patients, with specific haplotypes like HLA-DRB1*04:05:01 ~ DQB1*04:01:01 also present.^[8]^ Additionally, the HLA-DRB1*04 allele is associated with anti-citrullinated protein antibody (ACPA)-positive RA in Malaysian Chinese individuals.^[9]^ These findings suggest that relying solely on data from one Han subgroup can significantly underestimate the genetic heterogeneity of RA risk factors. Research on *HLA* polymorphisms in Nanning City, Guangxi Province, revealed that the local Han population has a closer genetic relationship with the Zhuang population than with northern Han populations, likely due to historical migration, environmental selection, and varying degrees of ethnic integration.^[10]^ Yunnan Province is a prime example of China’s ethnic diversity, situated at the core of a multi-ethnic convergence zone in southwest China. Since the Nanzhao Kingdom era, Yunnan’s Han population has extensively interacted with local ethnic groups, exchanged genetic material with Southeast Asian border communities, and coexisted with over 50 ethnic minorities. This complex ethnic blending suggests that Yunnan’s Han people may have unique genetic imprints, including gene traces from neighboring minorities like the Miao and Hani.^[11]^ These insights highlight the importance of detailed genetic research on Yunnan’s Han population and offer a unique model for analyzing the evolutionary trajectory of HLA molecules in cross-ethnic antigen responses.

Consequently, this study focuses on the Han population in Yunnan, China, systematically analyzing the association between *HLA-B* and *HLA-DR* gene co-polymorphisms and susceptibility to RA through a cross-sectional design for the first time. We hypothesize that there exists a specific relationship between the pathogenesis of RA in patients of the Yunnan Han nationality and *HLA-B/DRB1*. This relationship not only encompasses common susceptible genotypes found in RA patients but also includes unique genotypes. The objective is to elucidate the unique genetic risk profile of this population, provide region-specific data support for accurate typing and personalized treatment of RA, and establish critical benchmarks for assessing genetic risk related to RA among cross-border ethnic groups in Southeast Asia, such as Kokang in Myanmar and Daiyi in Vietnam.

## Patients and Methods

This study utilized a retrospective cross-sectional research design to include patients of Yunnan Han nationality diagnosed with RA who were admitted to the Department of Rheumatology and Immunology at the First Affiliated Hospital of Kunming Medical University between June 2017 and December 2018. Health controls (HCs) were recruited from the Health Management Center. All participants fulfilled the criteria related to regional genetic background, specifically having three generations of direct relatives belonging to the Han nationality. The ethical review was conducted in accordance with the principles set forth in the ‘Helsinki Declaration’. Furthermore, the research protocol received approval from the Ethics Committee of the First Affiliated Hospital of Kunming Medical University. All participants provided written informed consent.

### Inclusion and Exclusion Criteria

#### Inclusion Criteria of RA Patients Group

1. The patients met the “2010 American College of Rheumatology (ACR) / European League Against Rheumatism (EULAR) RA classification criteria” and were clinically diagnosed.^[12]^
2. Yunnan Han nationality, aged > 18 years.

#### Exclusion Criteria for RA Patients Group

1. Combined with other autoimmune diseases (such as systemic lupus erythematosus, Sjögren′s syndrome, *etc*.) or overlap syndrome.^[13]^
2. Active infection or receiving anti-infection treatment within 3 months.
3. Patients carrying known susceptibility genes associated with rheumatic and immunological diseases other than RA.

#### HCs Group Inclusion Criteria

1. All were healthy people of Yunnan Han nationality, aged > 18 years old.
2. All participants did not meet the 2010 ACR classification criteria for RA.
3. The HCs were matched with the RA patients for gender and age.
4. No family history of RA.

#### HCs Group Exclusion Criteria

Participants with any symptoms of joint swelling or pain, or any history of inflammatory joint symptoms.

### Data Collection and Sample Detection

In terms of clinical indicators, we collected data on autoantibodies, cytokines, erythrocyte sedimentation rate (ESR) and C-reactive protein (CRP) associated with RA. In addition, disease activity in RA patients was assessed using the DAS28-ESR (disease activity score based on 28 joints and erythrocyte sedimentation rate).

*HLA-B* and *HLA-DRB1* genotyping was conducted by high resolution reverse polymerase chain reaction sequence specific oligonucleotide probe (PCR-SSOP) in conjunction with Luminex xMAP^®^ liquid chip technology (Luminex 200, from ZEUS Company, USA). In this specific process, tailored primers were designed for the key regions of exon 2 and exon 3 polymorphisms of the *HLA-B* and *HLA-DRB1* genes to facilitate PCR amplification. Following purification, the resultant product was hybridized with biotin-labeled probes. The LABType® SSO HLA typing kit (Thermo Fisher Scientific PCR system 9700), which contains pre-coated oligonucleotide probes, was employed to detect fluorescence signal intensity *via* the Luminex system, thereby determining allele types.

Data analysis was performed using Luminex HLA Fusion 3.0 software, integrated with the IPD-IMGT/HLA database (v3.57) to validate typing results. The eight-digit naming convention was utilized to accurately differentiate subtypes (*e.g*., 04:05:01 *vs*. 04:05:02). For instances of typing ambiguity (*e.g*., *HLA-B15*: 01 *vs. B15:02*), verification was achieved through supplementary PCR-SSOP or Sanger sequencing methods to ensure that the minimum allele frequency detection threshold remained at 0.5%.

Haplotype analysis and result interpretation were accomplished by integrating reaction patterns alongside linkage disequilibrium characteristics of the *HLA-A/B/DRB1* linkage loci. Concurrently, stringent quality control standards were established; if internal control microbead signals did not meet threshold levels, experiments were deemed invalid, necessitating retesting or confirmation through alternative typing methodologies in cases of low signal or ambiguous results.

### Data Availability

A total of 246 patients diagnosed with RA and 259 gender and age-matched HCs were included in this study. Data on HLA-B alleles were collected from 246 RA patients, while data on HLA-DRB1 alleles were obtained from 234 patients. Additionally, information regarding both HLA-B and HLA-DRB1 alleles was gathered from the 259 HCs.

### Statistical Analysis

All experimental data were analyzed using IBM SPSS version 27.0 (USA). To assess the normality of quantitative data, we employed a single-sample Kolmogorov-Smirnov *Z* test. For measurement data that adhered to a normal distribution, results are presented as mean ± standard deviation, and an independent *t*-test was utilized to compare the means between two groups. In contrast, for data that did not conform to a normal distribution, the median (interquartile range) was used for intergroup comparisons, employing the Mann-Whitney *U* test accordingly. Categorical data were expressed as proportions, with either Pearson’s *chi-square* test or Fisher’s exact test applied for group comparisons.

## Result

### Demographic Characteristic

This study comprised 246 patients with RA (84% female, mean age 54.58 ± 11.72years) and 259 HCs (89% female, mean age 52.98 ± 9.63 years). The female-to-male ratio in RA group is 5.3. There were no statistically significant differences in the distribution of sex (*P* = 0.126) or age (*P* = 0.118) between the two groups (Table 1).

**Table 1:** Demographic and clinical characteristics of study participants.

	RA (*n* = 246)	HCs (*n* = 259)	*P*
Gender (female/male)	207/39	230/29	0.126
Age (yr)	54.58 ± 11.72	52.98 ± 9.63	0.118
Rheumatoid factor, IU/mL [M (Q1, Q3) ]	160.13 (7.05, 348.90)		
Negatives, *n* (%)	31 (12.60%)		
Low-titer positive, *n* (%)	32 (13.00%)		
High-titer positive, *n* (%)	183 (74.40%)		
Anti-CCP antibody, U/mL [M (Q1, Q3) ]	160.13 (0.60-1294.90)		
Negatives, *n* (%)	21 (8.50%)		
Low-titer positive, *n* (%)	17 (6.90%)		
High-titer positive, *n* (%)	208 (84.60%)		
DAS28-ESR			
≤ 2.6 (remission), *n* (%)	24 (9.80%)		
>2.6 to ≤ 3.2 (mild activity) *n* (%)	15 (6.10%)		
>3.2 to ≤5.1 (moderately activity) *n* (%)	106 (43.10%)		
>5.1 (severe activity) *n* (%)	101 (41.10%)		

RA, rheumatoid arthritis; HCs, healthy controls; anti-CCP antibody, anti cyclic citrullinated peptide antibody; DAS28-ESR, disease activity score based on 28 joints and erythrocyte sedimentation rate. Rheumatoid factor (<20 IU/mL: negative; 20-60 IU/mL: low-titer positive; >60 IU/mL: high-titer positive); Anti-CCP antibody (<20 U/mL: negative; 20-60 U/mL: low-titer positive; >60 U/mL; high-titer positive).

### Association of HLA-B Gene Polymorphism

A total of 110 *HLA-B* genotypes were identified among 492 alleles in a cohort of 246 RA patients, which was significantly higher than the 105 genotypes found in 518 alleles from 259 HCs. Notably, the allele frequencies for *B15:01:01G* (OR = 6.04, *P* = 0.001), *B40:01:01G* (OR = 2.51, *P* = 0.005), *B40:02:01G* (OR = 10.77, *P* = 0.024), and *B54:01:01G* (OR = 2.65, *P* = 0.005) were significantly elevated in the RA group. Conversely, the frequencies of the alleles *B15:01:01* (OR = 0.09, *P* = 0.019), *B15:02:01* (OR = 0.04, *P* = 0.012), and B*38:02:01 (OR = 0.35, *P* = 0.041) were significantly reduced in this RA patients group (Table 2).

**Table 2:** Comparison of alleles and haplotype with significant differences between HCs and RA

	RA (*n* = 492)			HCs (*n* = 518)	*P*	OR	95% CI
	*n*	carrier frequency (%)	*n*	carrier frequency (%)			
HLA-B allele	RA (*n* = 492)						
B*40:01:01G	32	6.5	14	2.7	0.005	2.51	1.33-4.77
B*40:02:01G	10	2.03	1	0.19	0.024	10.77	1.37-84.41
B*15:01:01G	22	4.47	4	0.77	0.001	6.04	2.07-17.65
B*54:01:01G	29	5.89	12	2.32	0.005	2.65	1.34-5.26
B*15:01:01	1	0.2	12	2.32	0.019	0.09	0.01-0.66
B*15:02:01	11	2.24	28	5.41	0.012	0.04	0.02-0.82
B*38:02:01	5	1.02	15	2.9	0.041	0.35	0.12-0.96
HLA‐DRB1 allele	RA (*n* = 468)		HCs (*n* = 518)				
DRB1*04:05:01	64	13.7	26	5.01	<0.001	3.02	1.88-4.86
DRB1*04:10:01	14	3	6	1.16	0.048	2.65	1.01-6.95
DRB1*10:01:02	17	3.64	7	1.35	0.025	2.77	1.14-6.74
DRB1*07:01:01	1	0.21	9	1.73	0.046	0.12	0.02-0.97
DRB1*14:01:01	2	0.43	23	4.43	0.001	0.09	0.02-0.40
DRB1*15:01:01:01	27	5.78	50	9.63	0.026	0.58	0.35-0.94
B gene type 1/DR gene type 1	RA (*n* = 468)		HCs (*n* = 512)				
B*15:01/DRB1*09:01	6	2.3	0	0	0.014	0.98	0.96-0.99
B*38:02/DRB1*08: 03	4	1.5	0	0	0.045	0.99	0.97-1.00
B gene type 2/DR gene type 2	RA (*n* = 468)		HCs (*n* = 512)				
B*54:01/DRB1*04:05	6	2.3	0	0	0.014	0.98	0.96-0.99

CI, confidence interval; RA, rheumatoid arthritis; HCs, health controls; OR, odds ratio.

### Polymorphism Characteristics of HLA-DR Gene

A total of 55 *HLA-DR* genotypes were identified among 468 alleles from 234 RA patients, which was significantly lower than the 60 genotypes found in 518 alleles from 259 HCs. The allele frequencies of DRB1*04:05:01 (OR = 3.02, *P* < 0.001), DRB1*04:10:01 (OR = 2.65, *P* = 0.048), and DRB1*10:01:02 (OR = 2.77, *P =* 0.025) were significantly elevated compared to those in the HCs group. Conversely, the allele frequencies of DRB1*07:01:01 (OR = 0.12, *P* = 0.046), DRB1*14:01:01 (OR = 0.09, *P* < 0.001), and DRB1*15:01:01:01 (OR = 0.58, *P* = 0.026) were notably reduced relative to the HCs group (Table 2).

### Haplotype Association Pattern

The haplotypes HLA-B15:01/DRB1*09:01 (*P* = 0.014), B38:02/DRB1*08:03 (*P* = 0.045), and B54:01/DRB1*04:05 (*P* = 0.014) were found to be significantly enriched in patients with RA (Table 2).

### Comparison of Anti-CCP Antibody Levels and DAS28-ESR in RA Patients with Diferent HLA-DRB1 Allele Carrying status

There was no significant difference in the levels of anticcp antibodies and DAS28-ESR between RA patients with shared epitope (SE) gene (DRB1*04:05:01, DRB1*04:10:01, and DRB1*10:01:02) carrying status and those without the carrying status (all *P* > 0.05)(Table 3).

**Table 3:** Comparison of anti-CCP antibody levels and DAS28-ESR in RA patients with different HLA-DRB1 allele carrying statuses (n = 468)

	DRB1*04:05:01		DRB1*04:10:01		DRB1*10:01:02	
	negative (*n* = 404)	positive (*n* = 64)	negative (*n* = 454)	positive (*n* = 14)	negative (*n* = 451)	positive (*n* = 17)
Anti-CCP antibody U/mL [*M* (Q_1_, Q_3_) ]	300.00 (122.08, 300.00)	300.00 (169.45, 300.00)	300.00 (123.52, 300.00)	297.18 (237.78, 300.00)	300.00 (122.32, 300.00)	300.00 (206.90, 300.00)
*Z*	-1.31		-0.19		-0.29	
*P*	0.19		0.848		0.775	
DAS28-ESR [*M* (Q_1_, Q_3_)]	4.82 (3.75, 5.89)	4.88 (3.98, 5.47)	4.81 (3.73, 5.78)	5.02 (4.17, 7.23)	4.81 (3.76, 5.79)	5.06 (4.71, 5.38)
*Z*	-0.37		-1.05		-0.47	
*P*	0.708		0.292		0.638	

Anti-CCP antibody, anti cyclic citrullinated peptide antibody; RA, rheumatoid arthritis; DAS28-ESR, disease activity score based on 28 joints and erythrocyte sedimentation rate; RA, rheumatoid arthritis.

## Discussion

In this study, we found that the proportion of women among Han RA patients in Yunnan was significantly higher, consistent with global trends showing increased female susceptibility to RA.^[5]^ The female-to-male ratio in our sample was similar to the prevalence in China.^[14]^ This higher prevalence in women may reflect regional differences or sample selection biases. The higher incidence of RA in females is likely due to interactions between X-linked chromosomes and hormonal factors, as seen in other autoimmune diseases.^[15]^

In our study, we identified risk associations with HLA-DRB1*04:05:01, *04:10:01, and *10:01:02 alleles in Yunnan Han Chinese RA patients. These alleles are all SE-encoded variants.^[16]^ Our findings are consistent with previous studies on RA patients from East China Han,^[8]^ Northern Han,^[17]^ and other East Asian regions ^[8]^ confirming the universality of the SE hypothesis across different ethnic groups. They also suggest that Yunnan Han RA patients share genetic features with the Han population in eastern China. However, unlike the DRB1*04:01/*04:04-dominant model in Caucasian populations.^[9,18]^ Our results show that the frequency of certain SE-encoding alleles varies among different populations.

HLA-DRB1*07:01:01, *14:01:01, and *15:01:01 alleles have been identified as protective against RA in the Yunnan Han population. The protective efects of HLA-DRB1*07:01 and *14:01 align with findings from Asian meta-analyses, while results for *15:01 show heterogeneity.^[19]^ Additionally, the impact of DRB1*14 on RA susceptibility is inconsistent across studies. Although it is associated with severe RA in South India,^[20]^ recent research suggests that DRB1*14 and *15 may protect against RA in Northeast India.^[21]^ However, these Indian studies did not distinguish between subtypes, highlighting the need for further research across diverse populations to clarify the specific roles of DRB1*14 and *15 subtypes in RA susceptibility.

There are limited studies investigating the association between *HLA* class I genes and susceptibility to RA, with significant correlations identified specifically for *HLA-B*.^[22]^ Previous research has indicated that a single nucleotide polymorphism (SNP) in the *HLA-B* locus, located at amino acid position 9 within the peptide binding groove, is associated with RA.^[23]^ In this study, we report for the first time that alleles HLA-B*40:01:01G, *40:02:01G, *15:01:01G, and *54:01:01G are susceptible genes for RA in the Yunnan Han population. Conversely, alleles HLA-B*15:01:01, *15:02:01, and *38:02:01 have been identified as protective genes against RA.

Notably, this study identified that the haplotypes HLA-B*38:02/DRB1*08:03, B*15:01/DRB1* 09:01 and HLA-B*54:01/DRB1*04:05 exhibited weak protective factors within the Yunnan Han RA population. The influence of HLA-DRB1*08:03 on RA susceptibility appears to be neutral; however, the protective effect associated with HLA-B*38:02/DRB1*08:03 may be contingent upon the specific genotype of HLA-B* 38: 02. Interestingly, while genotypes such as HLA-B*15:01, HLA-B*54:01, and HLA-DRB1*04:05 are recognized as risk factors for RA, their combination into haplotypes demonstrates a protective effect. This suggests that RA susceptibility is not solely dictated by individual *HLA* genotypes. There are limited studies on this cross-category (I/II) haplotype, and the findings have been inconsistent. The haplotypes mentioned above have been identified in the Han population for the first time, potentially reflecting unique genetic recombination events occurring in Yunnan. However, it is important to emphasize that, in addition to the interactions between *HLA* class I and II genes, certain associations within the major histocompatibility complex (MHC) region—including those involving *HLA* class III genes-must be understood through the lens of how closely linked genes function together as haplotype blocks.^[24]^

It is worth noting that this study did not find any significant differences between the SE allele carrying status and different anti-CCP antibody titers or disease activity (DAS28). This aligns with previous findings in China and reflects the relatively low prevalence of SE positivity in the Chinese RA population.^[25]^ Prior research has suggested that HLA-DRB1*04:05 may drive joint destruction by modulating the Th17/Treg balance rather than simply influencing ACPA production.^[26]^ This insight provides a novel perspective for understanding the clinical heterogeneity of RA.

The limitations of this study are as follows: (1) The detection efficiency is constrained by the sample size, particularly regarding low-frequency allele effects; (2) There is a lack of functional experiments to validate the biological mechanisms associated with key alleles; (3) The interaction analysis between *HLA-DQ/DP* alleles and non-*MHC* genes was not included. Future research should integrate high-depth *HLA* typing, epitope analysis, and organoid models to systematically investigate the molecular pathogenesis network of RA within the Chinese Han population. This is especially crucial for ACPA-negative subtypes, where it is necessary to explore the impacts of polymorphisms in *HLA-C*, *MHC* class I polypeptide-related sequence A (MICA), and other relevant genes.^[27]^

## Conclusion

This study reveals the unique *HLA* genetic profile of RA in Yunnan’s Han population, shaped by geography and historical migrations. It confirms race-adaptive variations linked to the SE hypothesis and identifies a new *HLA-I* risk model. The potential synergy between *HLA-I* and *HLA-II* could be a target for immune intervention, while functional analysis of protective alleles may inform precise prevention strategies. Compared to other regions, this research highlights regional differences in RA genetic susceptibility across China, enriching our understanding of RA’s genetic landscape. Comprehensive analysis of *HLA-B* and *HLA-DR* polymorphisms provides a complete picture of RA’s genetic factors. Correlating genetic findings with clinical outcomes *via* DAS28-ESR enhances the clinical relevance of this research. The study offers valuable references for future large-scale analyses and RA-targeted gene screening tools, and provides a basis for RA prevention and disease management strategies in Yunnan.
